# Effects of Periodontal Splints on Biomechanical Behaviors in Compromised Periodontal Tissues and Cement Layer: 3D Finite Element Analysis

**DOI:** 10.3390/polym14142835

**Published:** 2022-07-12

**Authors:** Yuchen Liu, Ming Fang, Ruifeng Zhao, Hengyan Liu, Min Tian, Sheng Zhong, Shizhu Bai

**Affiliations:** 1 State Key Laboratory of Military Stomatology, School of Stomatology, The Fourth Military Medical University, Xi’an 710032, China; 15294175510@163.com (Y.L.); zhaoruifeng0523@163.com (R.Z.); liu15137108609@163.com (H.L.); tianmin514718@163.com (M.T.); dczhongsheng@163.com (S.Z.); 2 National Clinical Research Center for Oral Diseases, School of Stomatology, The Fourth Military Medical University, Xi’an 710032, China; 3 Shaanxi Key Laboratory of Stomatology, School of Stomatology, The Fourth Military Medical University, Xi’an 710032, China; 4 Department of Prosthodontics, School of Stomatology, The Fourth Military Medical University, Xi’an 710032, China; 5 Digital Dentistry Center, School of Stomatology, The Fourth Military Medical University, Xi’an 710032, China

**Keywords:** periodontal splints, finite element analysis, polyetheretherketone, biomechanics

## Abstract

Background: In this study, we evaluated the effect of periodontal splints made from different materials on the stress distributions in compromised periodontal tissues and cement layers, using a computer simulation of mastication. Methods: Twenty-five 3D models were created for a segment of mandibular teeth with different periodontal splints bilaterally extended to the canines. The models were divided into five groups according to the different materials and thicknesses (mm) of the splints: the non-splinted group, PEEK 0.7 group, PEEK 1.0 group, FRC group, and titanium group. Each group was subdivided based on five bone loss levels. Tooth 41 of each model was subjected to vertical and oblique (θ = 45°) static loads of 100 N, respectively, onto the incisal edge. The von Mises stresses and maximum principal stress were analyzed using Abaqus software. Results: Oblique loading resulted in higher stresses on periodontal tissues, cement layers, and splints than those caused by vertical loading. The lower the supporting bone level, the greater the stress difference between the splinted groups and the non-splinted group. In model 133,331, with severe bone loss, the maximum von Mises stress values on the alveolar bone in tooth 41 under oblique loading dramatically decreased from 406.4 MPa in the non-splinted group to 28.62 MPa in the PEEK group and to 9.59 MPa in the titanium group. The four splinted groups presented similar stress distributions in periodontal tissues. The lowest stress level on the splint was observed in the PEEK 0.7 group, and the highest stress level was transferred to the cement layer in this group. Stress concentrations were primarily exhibited at the connectors near the load-carrying area. Conclusions: The tested splinted groups were all effective in distributing the loads on periodontal tissues around splinted teeth with similar patterns. Using splinting materials with low elastic moduli reduced the stress concentration at the splint connectors, whereas the tensile stress concentration was increased in the cement layer. Thus, the use of adhesive cement with a higher elastic modulus is recommended when applying less rigid PEEK splints.

## 1. Introduction

Periodontitis is a microbially associated, host-mediated inflammatory disease. Its primary features include the loss of periodontal attachment and marginal alveolar bone loss [1]. Progressive alveolar bone loss eventually leads to hypermobility of the teeth, which has a negative impact on masticatory function or chewing comfort, aesthetics, and phonetics [2]. Efforts should be directed toward tooth splinting for patients with mobile teeth [3]. Splinting is defined as connecting pathological mobile teeth with relatively healthy adjacent ones into a rigid unit, which is commonly used in mandibular anterior teeth. Such a fixed unit can not only stabilize mobile teeth in a predetermined position to restore patients’ psychological and physical well-being but also decrease stress concentrations [2,3]. In addition, splinting can induce bone-remodeling processes, consequently, preventing bone loss [4]. 

Regarding materials used in dental immobilization systems, fiber-reinforced composite has been used for a long time due to its high strength, pliability, bondability, biocompatibility, and esthetics. However, it requires lengthy chair-side procedures. In addition, discoloration and dislodgement are possible complications [5]. Moreover, fiber-reinforced composite resin easily induces plaque formation, which has an adverse effect on periodontal maintenance.

Computer-aided design/computer-aided manufacturing (CAD/CAM) technology is one of the fastest-evolving aspects of modern dentistry, offering some improvements to periodontal splints. First, it can increase patients’ comfort and improve cooperation [6]. In addition, CAD/CAM-processed indirect periodontal splints have a closer fit with the curved dental arch and lingual teeth profile than direct ones [7]. Most importantly, the need for time-consuming clinical procedures is reduced. CAD/CAM technology makes it possible for dentists to perform these procedures more accurately and efficiently. 

Two main types of materials currently available for use in CAD/CAM splints are titanium and polyetheretherketone (PEEK) [7,8]. PEEK is a thermoplastic, tooth-colored polymetric material, with extreme heat resistance and high performance. Due to its excellent physical and biological properties, and the fact that it has better esthetics than metals, PEEK has been proposed for use in prosthodontic applications, such as in implants, provisional abutments, crowns, bridges, and removable partial dentures [9,10]. Another positive feature of PEEK is its low plaque affinity, which is important when treating patients with periodontitis. An in vitro study revealed that the formation of dental biofilm on the PEEK surface is even lower than that on titanium and zirconia ceramics [11]. Thereby, it seems that PEEK is advantageous compared with titanium in the fabrication of periodontal splints. 

The clinical longevity of periodontal splints depends to a great extent on their biomechanical behaviors in compromised periodontal tissues and cement layers. Different periodontal splints exhibit different patterns of load distribution and transfer, due to the different stiffnesses in splinting materials [2,4]. An understanding of stress magnitude and distribution in reduced periodontal supporting tissues is crucial to avoid the major complications in periodontal splints: debonding and fracture [2,4]. However, there is little biomechanical evidence to compare the effectiveness of PEEK splints with that of other periodontal splints. Finite element analysis (FEA) is a non-invasive tool for use in the study of biomechanics and the effect of mechanical strength on biological systems [12]. In dentistry, FEA has been used to estimate stress distribution in periodontium [13]. As the periodontal ligament (PDL) is the most crucial component of periodontium, the inclusion of the PDL in an FEA model yields more reliable results. Although a model of this type may not completely replicate the oral environment, the results may be referred to qualitatively [12].

Therefore, the aim of this study was to evaluate the effect of four types of splints under loading on stress distributions in periodontal tissues, cement layers, and splints themselves, and to assess the impact of PEEK splints in terms of both the risk of debonding from abutment teeth and damage to periodontal tissues. 

## 2. Materials and Methods

### 2.1. Generation of the Geometric Models and Study Design

Computed tomography scans of the lower jaw and mandibular dentition were obtained from a Chinese patient with full natural dentition. The experimental procedures were approved by the Medical Ethics Committee of the School of Stomatology, the Fourth Military Medical University (Reference number IRB-REV-2021134). We collected a total of 640 slices and saved them as DICOM files. Image data sets were processed via 3D image generation software Mimics Research 19.0 (Materialise NV, Leuven, Belgium). The mandibular structures were segmented as bone and teeth using an image density thresholding tool, which isolates different structures by converting grayscale values into binary images. Incisors, canines, first premolars, and second premolars were separated from each other. Then, the preliminary models were polished to obtain smooth models with no irregularities, and the data were converted to the STL. file format.

The volumetric models, including those of mandible and segment teeth, were imported into Geomagic Wrap 2015 (3D Systems, Rock Hill, SC, USA). The bone was segmented as a cancellous core surrounded by a cortical layer. The PDL was virtually constructed around the tooth roots based on their actual anatomy. Periodontal splints that extended to the canines bilaterally and their cement layer were molded based on the lingual anatomy of the abutment teeth. Resin cement with a thickness of 0.1 mm was used for the cement layer as it is commonly used clinically. The models were then divided into five groups according to the different materials and thicknesses (mm) of the splints: the non-splinted group, PEEK 0.7 group, PEEK 1.0 group, fiber-reinforced resin (FRC) group, and titanium group. All materials and structures were assumed to be homogeneous, isotropic, and linearly elastic. The Young’s modulus and Poisson’s ratios were assigned according to values from the literature (Table 1). All these parts were converted into non-uniform rational B-spline surfaces and saved as Initial Graphics Exchange Specification (IGES) files. These IGES files were then imported into the multi-disciplinary finite element pre-processor software HyperMesh 19.0 (Altair Engineering, Troy, MI, USA).

**Figure 1:**
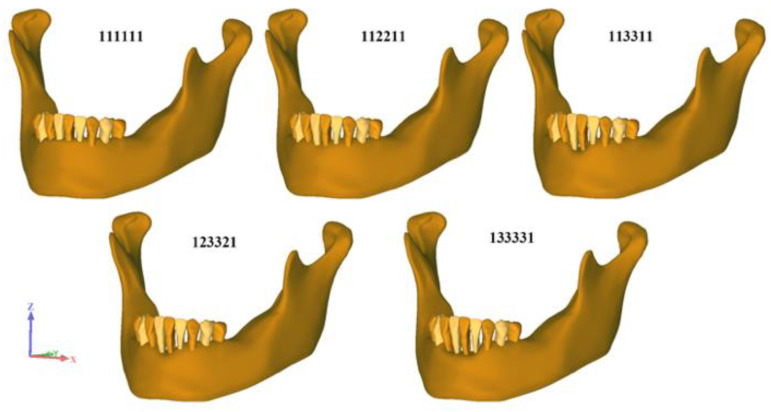
Different levels of horizontal bone loss.

In order to simulate various clinical scenarios regarding the levels of horizontal bone loss, five different geometries of periodontal tissues were used (Figure 1 and Table 2).

### 2.2. Finite Element Analysis (FEA)

**Figure 2:**
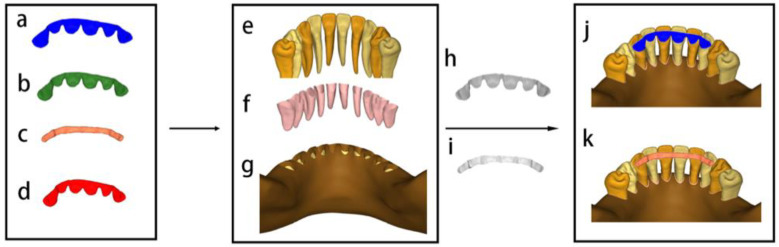
Schematic illustration of the modelled structures used in this study. Different materials were considered for periodontal splinting: (**a**) PEEK splint with 0.7 mm thickness, (**b**) PEEK splint with 1.0 mm thickness, (**c**) FRC splint, and (**d**) titanium splint. Mandible segmentation contained: (**e**) premolars, canines, lateral incisors, and incisors, (**f**) periodontal ligament, (**g**) cortical bone and spongy bone. The final models with periodontal splinting positioned on the anterior teeth (**j**,**k**): (**h**) cement layer of PEEK or titanium splint, (**i**) cement layer of FRC.

Uniform surface meshes and isoparametric tetrahedral elements with increasing mesh densities were used until convergence was achieved using HyperMesh software. The element used in the mesh division was tetrahedral with 10 nodes (Tet-10). A mesh convergence test was performed, determining a finite number of 1,468,572~1,646,534 elements and 335,005~371,127 nodes. PEEK and titanium splints shared one type of cement layer. Due to the different geometry of FRC, a strip-shaped cement layer was used in the FRC group. Then, all the models were imported into the Abaqus/CAE 2019 software (SIMULIA, Dassault Systèmes, Vélizy-Villacoublay, France) for stress analysis (Figure 2). 

Contact was defined using surface-to-surface discretization for more accurate stress results compared with node-to-surface discretization [14]. Tie constraints were set up between the bone, periodontal ligament, cement layer, periodontal splint, and teeth. Such contact could avoid relative motion and any friction. The adjacent teeth interproximal surfaces in periodontal compromised tissues were lost, which did not lead to existing stress transmission from one element to another at the interface region. Therefore, the relationship between teeth elements was not defined. 

As mentioned above, a total of 25 models were developed. In order to simulate biting forces, each model was subjected to vertical and oblique (θ = 45°) static loads of 100 N, respectively, from the midpoint of the incisal edge on tooth 41. The fixed support was defined on the condylar, coronoid, and inferior border of the mandible, in case of any displacement or rotation in the models. 

The equivalent von Mises stress magnitudes and distributions in the PDL and the alveolar bone, as well as the splints themselves were measured through colorimetric graphs in all the groups, and the maximum principal stress in the cement layer was calculated. The material failed when the equivalent von Mises stress values exceeded the tensile strength of the material [19]. Due to the numerical nature of this work and the fact that additional series of observations would yield the same data, the statistical analysis could not be performed.

## 3. Results

### 3.1. Maximum von Mises Stress on Periodontal Tissues in Non-splinted Models with Different Bone Levels

**Figure 3:**
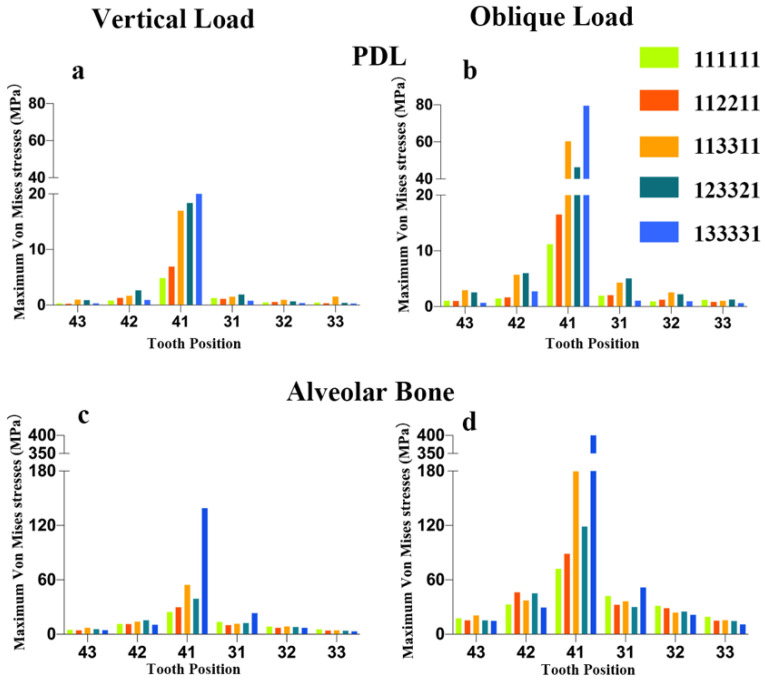
Maximum von Mises stress (MPa) on PDL and alveolar bone in non-splinted group with different bone levels: (**a**) stress on PDL in non-splinted groups under vertical loading, (**b**) stress on PDL in non-splinted groups under oblique loading, (**c**) stress on alveolar bone in non-splinted groups under vertical loading, (**d**) stress on alveolar bone in non-splinted groups under oblique loading.

As shown in Figure 3, the maximum von Mises stress values on the PDL and alveolar bone increased with reduced bone levels around tooth 41 and adjacent teeth in non-splinted models, especially under oblique loading. Only a small amount of the load on tooth 41 was transferred to the alveolar bone of the adjacent teeth 42 and 31, which did not have periodontal splinting.

### 3.2. Maximum von Mises Stress on Periodontal Tissues Surrounding Splinted Teeth with Different Bone Levels

**Figure 4:**
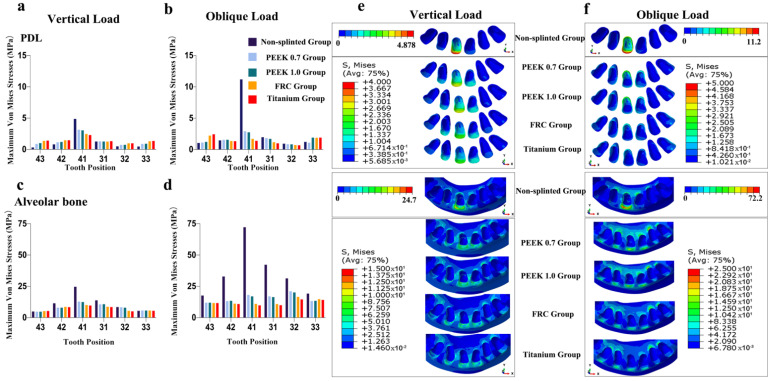
Maximum von Mises stresses and occlusal views of stress distributions in periodontal tissues of different groups for model 111111: (**a**) stress on PDL under vertical loading, (**b**) stress on PDL under oblique loading, (**c**) stress on alveolar bone under vertical loading, (**d**) stress on alveolar bone under oblique loading, (**e**) stress distributions in PDL and alveolar bone under vertical loading, and (**f**) stress distributions in PDL and alveolar bone under oblique loading.

**Figure 5:**
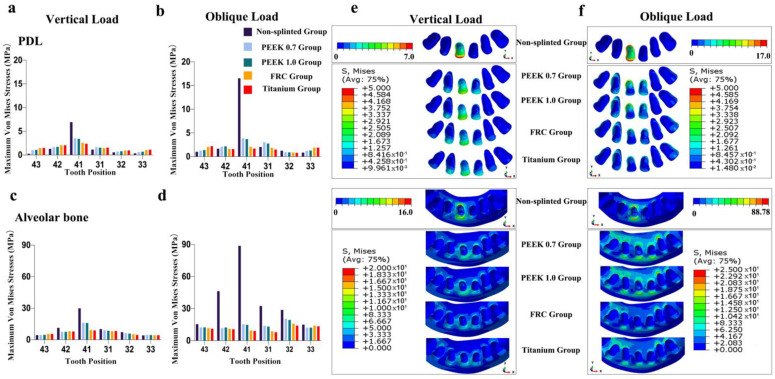
Maximum von Mises stresses and occlusal views of stress distributions in periodontal tissues of different groups for model 112211: (**a**) stress on PDL under vertical loading, (**b**) stress on PDL under oblique loading, (**c**) stress on alveolar bone under vertical loading, (**d**) stress on alveolar bone under oblique loading, (**e**) stress distributions in PDL and alveolar bone under vertical loading, and (**f**) stress distributions in PDL and alveolar bone under oblique loading.

**Figure 6:**
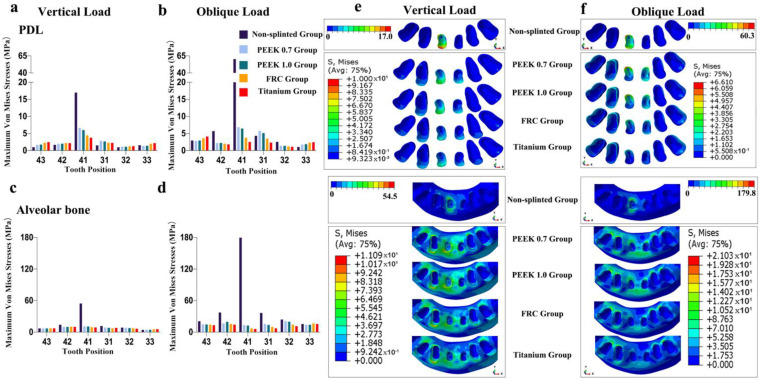
Maximum von Mises stresses and occlusal views of stress distributions in periodontal tissues of different groups for model 113311: (**a**) stress on PDL under vertical loading, (**b**) stress on PDL under oblique loading, (**c**) stress on alveolar bone under vertical loading, (**d**) stress on alveolar bone under oblique loading, (**e**) stress distributions in PDL and alveolar bone under vertical loading, and (**f**) stress distributions in PDL and alveolar bone under oblique loading.

**Figure 7:**
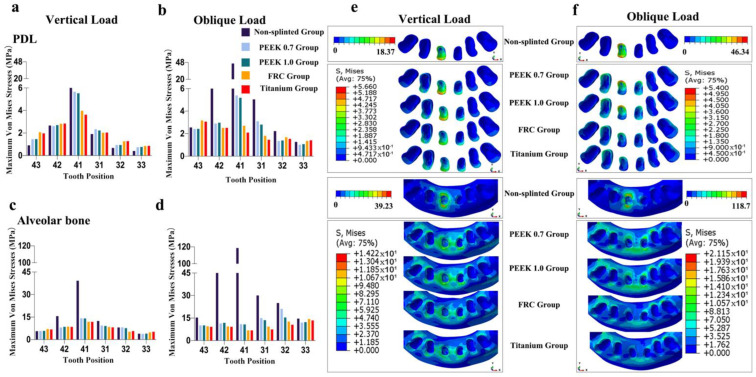
Maximum von Mises stresses and occlusal views of stress distributions in periodontal tissues of different groups for model 123321: (**a**) stress on PDL under vertical loading, (**b**) stress on PDL under oblique loading, (**c**) stress on alveolar bone under vertical loading, (**d**) stress on alveolar bone under oblique loading, (**e**) stress distributions in PDL and alveolar bone under vertical loading, and (**f**) stress distributions in PDL and alveolar bone under oblique loading.

**Figure 8:**
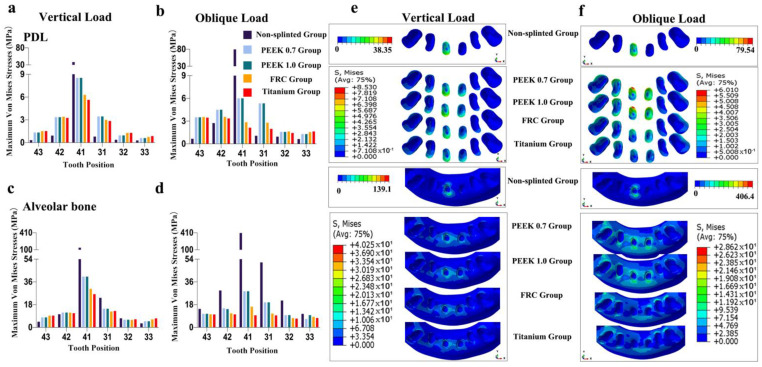
Maximum von Mises stresses and occlusal views of stress distributions in periodontal tissues of different groups for model 133331: (**a**) stress on PDL under vertical loading, (**b**) stress on PDL under oblique loading, (**c**) stress on alveolar bone under vertical loading, (**d**) stress on alveolar bone under oblique loading, (**e**) stress distributions in PDL and alveolar bone under vertical loading, and (**f**) stress distributions in PDL and alveolar bone under oblique loading.

As shown in Figure 4, Figure 5, Figure 6, Figure 7 and Figure 8, the von Mises stress value on the alveolar bone was higher than that on the PDL, regardless of the model and loading condition. The lower the supporting bone level, the greater the stress difference between the splinted groups and the non-splinted group. Four splinted groups presented similar stress magnitudes and distribution patterns on periodontal tissues.

The maximum von Mises stress values on periodontal tissues in tooth 41 in the non-splinted group were higher than those in the splinted groups, among which stresses were slightly higher in the PEEK groups than those in the FRC and titanium groups. In particular, in model 133331 with severe bone loss exhibited in Figure 8, the maximum von Mises stress values on the alveolar bone in tooth 41 under oblique loading dramatically decreased from 406.4 MPa in the non-splinted group to 28.62 MPa in the PEEK group and to 9.59 MPa in the titanium group. In addition, periodontal splinting was shown to distribute slightly higher stress values to the PDL in the canines than those in non-splinted ones. Under vertical loading, splinted groups presented similar stress distribution patterns in canine alveolar bone. However, there was no notable difference in the maximum von Mises stress values among groups under oblique loading.

### 3.3. Maximum Principal Stresses on the Cement Layer and Maximum Von Mises Stresses on the Periodontal Splints

**Figure 9:**
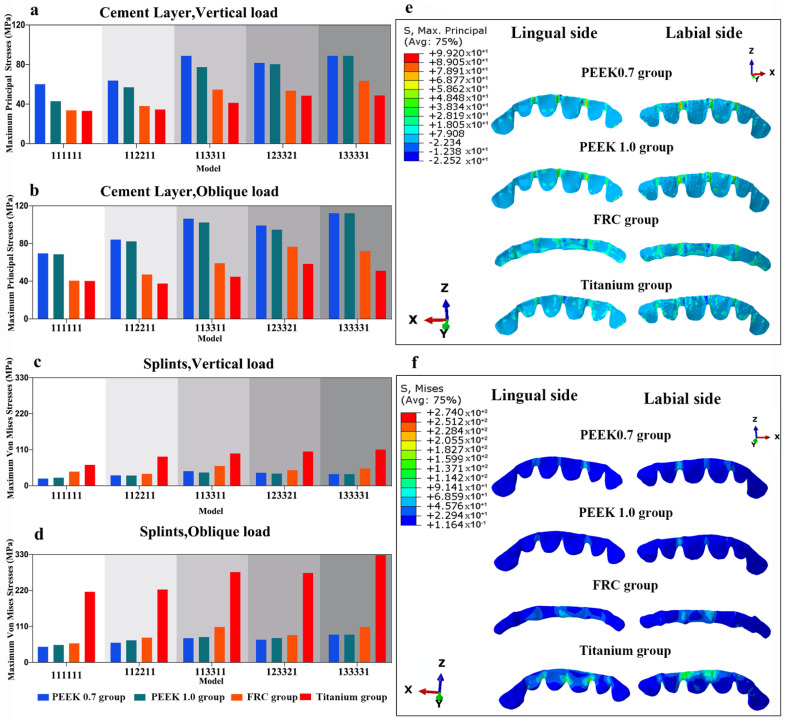
Stresses of different groups and their stress distributions in model 123321: (**a**) maximum principal stresses on cement layer under vertical loading, (**b**) maximum principal stresses on cement layer under oblique loading, (**c**) maximum von Mises stresses on periodontal splints under vertical loading, (**d**) maximum von Mises stresses on periodontal splints under oblique loading, (**e**) principal stresses distribution in cement layer under oblique loading in model 123321, and (**f**) principal stresses distribution in periodontal splints under oblique loading in model 123321.

The magnitude and distributions of stress on the cement layers and different periodontal splints are displayed in Figure 9.

It could be observed that the greater the elastic modulus of the splint, the higher the stress value that was concentrated on it, and the lower the stress value that was transferred to the cement layer. The highest stress value on the splint was observed in the titanium group, and the lowest stress value was transferred to the cement layer in this group. Oblique loading resulted in higher stress values than vertical loading in all the groups. More severe alveolar bone loss induced augmented stress on splints.

Among all the groups, the stress concentrations in the cement layer and splints were primarily exhibited at the connectors near the load-carrying area. 

## 4. Discussion

Horizontal bone loss is one of the consequences of chronic periodontitis. In the present study, it was demonstrated that the stress values on the remaining PDL and alveolar bone increased with reduced bone levels in non-splinted mandibular anterior teeth. Other studies have shown a similar trend [20,21,22,23]. It has been shown that reduced alveolar bone support leads to unfavorable crown to root ratios in the abutment teeth, which can increase the leverage. Under this circumstance, abnormal stress values concentrate in the periodontium, especially in the alveolar bone crest [24]. The accumulation of stress in the alveolar bone may lead to the exacerbation of the situation in the defect region and possibly induce further bone resorption [24]. In addition, compromised alveolar bone support results in a reduced PDL volume. This may be one of the reasons why the stress values in PDLs may rise [22]. The presence of the PDL makes it possible to distribute and absorb forces produced during mastication [25]. Reduced PDL volume may cause secondary occlusal trauma [26]. The simultaneous occurrence of periodontitis and occlusal trauma could accelerate pre-existing periodontal lesions [27]. Therefore, it is necessary to reduce the deteriorating effects of compromised supporting tissues under physiologic loads.

Splints are commonly used to reduce the risk of secondary traumatic occlusion on periodontally compromised teeth and facilitate the distribution of occlusal forces [2]. Our research showed that splinting abutment teeth with different materials led to a dramatic decrease in stress values in periodontal tissues. This is in line with a study conducted by Soares et al., who demonstrated that the main objective of stabilizing teeth with a splint is the reduction in biomechanical stresses in the supporting bone structure [21]. 

As the maximum occlusal load on incisors was reported to vary between 40 and 200 N [28], a concentrated 100-N load was regarded to be within physiologic limits and was applied vertically or obliquely in the current study. The oblique load was applied at an angle of 45° to simulate the contact of the mandibular incisal edges with the lingual surfaces of the maxillary teeth [7,20,21]. Although the occlusal loads in the mandibular anterior region are relatively small and distributed evenly, concentrated occlusal force can be produced under conditions such as a hard bite into food. Oblique loading resulted in higher stress values on periodontal tissues, cement layers, and splints than those caused by vertical loading, which is in accordance with previous studies [29,30]. The highest equivalent stress may lead to periodontal hazards [13,30]. Emphasis should be placed on the importance of avoiding or reducing oblique loading.

Young’s modulus (an intrinsic mechanical property of the material) is an indicator of material stiffness and illustrates a positive association between the modulus of elasticity and substance rigidity [31]. Rigid materials tend to have high elastic moduli. Splints with different materials have different patterns of transmission and cause different distributions of masticatory forces. This is the reason why they have different biomechanical responses in periodontal tissues [21]. Although current guidelines for the treatment of mobile teeth and traumatic injuries recommend the use of ‘flexible’ splints, the specific definition of what is considered flexible versus rigid has not been clearly defined [2]. Among the tested materials in the current study, PEEK is the most flexible splint in terms of its elastic modulus, followed by fiber-reinforced composite, and titanium is the most rigid. The PEEK splints we used here were made from polymer-BioHPP, which adds 20% special ceramic filler to pure PEEK [32]. The fiber-reinforced composite splints were simulated virtually with Quartz Splints, containing silica fibers. Silica in silica fibers is crystalline, whereas it is amorphous in glass fibers. Silica fiber exhibits higher flexural strength than glass fiber [33]. A previous study carried out by Saquib et al. showed that polyethylene fibers are more flexible than glass fibers [31]. Ribbond^®^ is made from leno weave, an ultra-high-molecular-weight polyethylene fiber, which is commonly used for splinting [2]. Flexible splinting may recover the physiologic mobility of teeth and thus may foster bone-remodeling stimulation in a previously jeopardized periodontium [31]. However, rigid splints may limit the movement of abutment teeth. An in vitro study claimed that mild tooth movement would favor the revascularization process, whereas complete immobilization would interfere with fibroblast metabolism [34]. This is the reason why flexible splinting is recommended. In our investigation, the greater the stiffness of the splinting material tested, the lower the maximum von Mises stress value that was concentrated on the alveolar bone. PEEK groups induced higher stress values on the periodontal structures, but it remains unclear whether such stress values would lead to bone loss or bone remodeling. Further laboratory studies are still needed to clarify the issue, as alveolar bone is a dynamic material, the properties of which are influenced by a number of factors. Theoretically, PEEK splints can allow the physiological movement of the mobile abutment teeth, which may promote improved healing of the periodontal tissues compared to rigid splints. Ribbond^®^, the elastic modulus of which (4 GPa) is comparable to that of PEEK [31], has been proven to be an excellent material for splinting mobile teeth due to its flexibility [2,35]. However, during the masticatory process, the infiltrating composite resin inside Ribbond^®^ may undergo microcracks and expansion. In addition, direct splinting increases the chair-side time and needs complex manipulation. CAD/CAM PEEK blocks can solve the abovementioned problems, and thus may be a potential alternative to Ribbond^®^ for periodontal splinting. Although it may require more laboratory research, the use of PEEK for periodontal splinting has many advantages, such as reducing the need for time-consuming clinical procedures, better adaptation to abutment, etc.

Despite the fact that splinted teeth can survive for a long time, significant efforts are necessary to retain these teeth [36]. A high number of repairs are needed as splint fracture or debonding was documented in 25.6% of patients within the first 3 years after direct periodontal splinting [37]. Thereby, finding measures to prevent splint fracture or debonding is a critical issue to improve the clinical longevity of periodontal splints. 

When occlusal force is loaded on abutment teeth, the splint deforms, and peel force is generated in the cement layer, promoting the debonding of the splint. It was revealed in our research that high stress concentrations were primarily exhibited at the splint connectors near the load-carrying site, which is the high-risk area for debonding and should be thickened. With regard to maximum principal stress on the cement layer among all models, more stresses were induced in the PEEK 0.7 group compared with the FRC group and titanium group. This finding corroborates with those of previous studies, in which rigid restorative materials presented reduced stresses on the cement layer [38,39], whereas a flexible prosthesis showed higher displacement than a rigid one, inducing a higher stress concentration at the adhesive interface [40]. In this way, when applying a less rigid PEEK splint, the use of adhesive cement with a higher elastic modulus is recommended, as it can concentrate less tensile stress at the connectors [41]. 

The fracture resistance of a restoration depends on the strength of the material from which it is fabricated. In our investigation, it was found that the greater the elastic modulus of the splint, the higher the level of stress that is concentrated on it. Due to its relatively lower elastic modulus, the lowest stress on the splint was observed in the PEEK 0.7 group. The tensile strength of PEEK is 100.69 MPa [42]. The maximum von Mises stresses calculated in this study ranged from 22.07 MPa to 88.15 MPa, indicating that PEEK splints in mandibular anterior teeth may have good fracture resistance. Nevertheless, a number of factors are associated with the long-term behavior of splints. We found that more severe alveolar bone loss induced augmented stress on splints. This is in line with the finding of a clinical study, which showed that teeth with more severe bone loss presented lower resistance to chewing forces, and the resulting stress onto the splint could lead to a higher likelihood of splint fractures [36]. Long-term clinical observations are needed to further evaluate the clinical performance of PEEK splints. 

FEA is a highly valuable research tool [26]. Nevertheless, there are still limitations to this type of study. Not all factors were numerically simulated in the analysis, due to the complexity of the dynamic oral cavity and its internal biological phenomena. Complete and permanent bonding depends on multiple factors in addition to stress concentration. Cyclic loading and the fatigue and endurance of the splints were not studied in the present study. Mathematical analysis generates theoretical results which need to be correlated with laboratory and clinical findings. The long-term clinical performance of different splinting materials should be evaluated in future studies.

## 5. Conclusions

Within the limitations of the current finite element analysis, in conclusion, oblique loading resulted in higher stress values on periodontal tissues, cement layers, and splints than those caused by vertical loading. The stresses on the remaining periodontal ligament and alveolar bone increased with the severity of bone loss. The tested splinted groups were all effective in distributing the loads on periodontal tissues around splinted teeth with similar patterns. The use of splinting material with a low elastic modulus, such as PEEK, reduces the stress concentration at the splint connectors while increasing the tensile stress concentration in the cement layer. 

## Figures and Tables

**Table 1:** Mechanical properties of the materials/structures applied in models of mandibular anterior teeth splinted with different materials.

Material/Structure	Thickness (mm)	Young’s Modulus (GPa)	Poisson’s Ratio	References
PEEK	1.0/0.7	4.1	0.45	[9]
FRC	1.0	37	0.3	-
Titanium	1.2	110	0.35	[14]
Resin cement	0.1	7.3	0.3	[15]
Tooth	-	18.6	0.3217	[16]
Cortical bone	2	13.7	0.318	[14,17]
Spongy bone	-	1.37	0.3	[17]
Periodontal ligament	0.2	0.069	0.45	[17,18]

**Table 2:** Model description, number of elements and nodes for each model.

Model	Bone Level (%)						
	Tooth	43	42	41	31	32	33
111111		75	75	75	75	75	75
112211		75	75	50	50	75	75
113311		75	75	30	30	75	75
123321		75	50	30	30	50	75
133331		75	30	30	30	30	75

## Data Availability

The data presented in this study are available upon request from the corresponding author.
